# Hypercortisolemia Recurrence in Cushing's Disease; a Diagnostic Challenge

**DOI:** 10.3389/fendo.2019.00740

**Published:** 2019-11-08

**Authors:** José Miguel Hinojosa-Amaya, Elena V. Varlamov, Shirley McCartney, Maria Fleseriu

**Affiliations:** ^1^Departments of Medicine (Endocrinology), Oregon Health & Science University, Portland, OR, United States; ^2^Endocrinology Division, Department of Medicine, Hospital Universitario “Dr. José E. González”, Universidad Autónoma de Nuevo León, Monterrey, Mexico; ^3^Neurological Surgery, Oregon Health & Science University, Portland, OR, United States; ^4^Northwest Pituitary Center, Oregon Health & Science University, Portland, OR, United States

**Keywords:** hypercortisolemia, recurrence, Cushing's disease, Cushing's syndrome, Cushing, diagnostic testing, remission

## Abstract

Cushing's disease recurrence following successful pituitary surgery is common and merits prompt and careful diagnosis, as untreated hypercortisolism leads to increased morbidity and mortality. However, an established recurrence definition has not been forthcoming. This poses a diagnostic challenge especially early in the course of returning hypercortisolemia and/or in the presence of non-neoplastic hypercortisolemia. A late-night salivary cortisol (LNSC) test is the first test to reveal abnormal results, however, has limitations related to assay performance as well as individual patient variability. Dexamethasone suppression tests and 24-h urinary free cortisol (UFC) results are next to reveal abnormal results. Other tests including, corticotropin-releasing hormone (CRH) stimulation test and combined CRH-dexamethasone test, as well as desmopressin stimulation test with/without dexamethasone are also used, although, none have proven to be the preeminent diagnostic test for recurrence determination. There is a possible role for these tests in predicting recurrence in patients who have experienced remission, though, this also remains challenging due to lack of established cutoff values. This article details and summarizes evidence about different diagnostic tests currently used to diagnose and predict Cushing's disease recurrence.

## Introduction

Endogenous Cushing's syndrome (CS) is caused in ~80% of cases by an adrenocorticotropic hormone (ACTH)-producing pituitary adenoma (Cushing's disease; CD), in other cases by a cortisol-producing adrenal source (adrenal adenoma, hyperplasia, or carcinoma) and in less frequent cases an ectopic ACTH-secreting neuroendocrine tumor (1–4).

Hypercortisolemia is associated with changes in body composition and metabolic comorbidities such as dyslipidemia, insulin resistance, diabetes mellitus, hypercoagulability, and hypertension (1–4). Untreated CS, especially in severe cases, has a poor prognosis and high mortality, with patient survival rates of only 50% after 5 years (5) while treated disease has a much better prognosis (3). Excess mortality derives from cardiovascular complications such as myocardial infarction and stroke, however, uncontrolled diabetes, and opportunistic infections also play a major role in impaired patient survival (3, 6). Recent meta-analysis data showed that although mortality remains significantly increased after remission by standardized mortality ratios (SMR) of 2.5 compared to general population, mortality risk in the uncontrolled disease population is even higher (SMR 4.6) compared with patients in remission (SMR 1.8, 95% CI 0.95–3.7) (3). As such, the importance of establishing guidelines for timely CS recurrence diagnosis is paramount.

Remission rates for CD following transsphenoidal surgery (TSS) vary between 42 and 90% and are considerably lower in macroadenoma cases (1, 2). Recurrence after initially successful TSS may occur in up to 66% of cases, with a higher rate in macroadenomas cases (7). Pituitary centers that perform a large number of pituitary surgeries have better outcomes, less morbidity, and mortality (8–10), however, surgical skills and the definition of either remission or recurrence used in studies, which can vary greatly, influence results. Various studies on rates of CD remission using different criteria have been undertaken and a summary is shown in Supplemental Table 1. Study correlations between different markers of hypercortisolism are limited. One study assessing variability of late-night salivary cortisol (LNSC) in 19 patients (8 with *de novo* and 8 with recurrent CD) showed a poor correlation between 24-h urinary free cortisol (UFC) and LNSC (Pearson correlation coefficient *R* = 0.419; *p* = 0.15) (11), while a larger study in 93 patients treated with pasireotide found a moderate correlation between LNSC and 24-h UFC (10).

Once hypercortisolemia is noted in a patient with suspected CD recurrence, other potential causes of non-neoplastic hypercortisolism (i.e., acute psychological or physical stress, obesity, depression, chronic excessive alcohol use) should be excluded before a diagnosis is confirmed, however, in most cases, the unequivocal finding of hypercortisolemia in a patient previously diagnosed with CD favors a diagnosis of recurrence. However, as 24-h UFC is the last test to reveal abnormal results, a 3 to 4-fold elevation over the upper limit of normal (ULN) alleviates the need for further work-up (2, 12, 13). Studies on CD recurrence rates using different criteria have been undertaken and a summary is shown in Table 1.

**Table 1:** Studies by year (2001–2019) on the criteria for Cushing's disease recurrence.

**Year** **Author**	**No. of** **patients**	**Recurrence** **criteria**	**Recurrence** **(%)**	**Mean or median** **time to recurrence** **(range), months**
**2001**				
1. Barbetta	68	Clinical SC LDDST	21	NA
2. Cavagnini	288	Clinical SC UFC	15	NA
3. Chee	61	Clinical SC UFC	14.6	76.1 (22–158)
**2002**				
1. Rees	53	Clinical Morning SC UFC	5	(13–36)
2. Shimon	74	Clinical SC LDDST UFC	5.2	(24–60)
3. Yap	97	LDDST UFC	11.5	36.3 (6–60)
**2003**				
1. Chen	174	Clinical SC UFC	NA	(6–48)
2. Flitsch	147	Clinical SC	5.6	NA
3. Pereira	78	Clinical LDDST UFC	9	84
**2004**				
1. Hammer	289	Clinical SC LDDST UFC	8.7	58.5 (13.2–133.2)
2. Rollin	48	Clinical SC LDDST	4.2	(54–66)
3. Salenave	54	Morning SC UFC	19.5	NA
**2005**				
1. Atkinson	63	Clinical SC LDDST UFC	22.2	63.6 (12–108)
**2006**				
1. Esposito	40	Clinical SC	3.1	NA
2. Hofmann	100	Clinical SC LDDST	4.8	18.8 (3–86)
**2007**				
1. Acebes	44	Clinical Morning SC UFC	7.7	54.6 (30–84)
2. Rollin	103	Clinical SC LDDST	6.8	(24–66)
**2008**				
1. Hofmann	426	Clinical SC LDDST	15	NA
2. Jehle	193	Morning SC LDDST UFC	13.5	57.6 (8.4–148.8)
3. Patil	215	Clinical UFC	17.4	39 (3–134)
4. Prevedello	167	Clinical UFC	12.8	50 (12–117)
5. Carrasco	68	Morning SC LDDST UFC	14.3	51 (9–90)
**2009**				
1. Castinetti	38	UFC Night ACTH Night cortisol LDST CDDT	26.30%	NA
2. Fomekong	40	Clinical UFC	9.4	NA (18–96)
3. Jagannathan	261	Clinical	2.3	56 (5–129)
4. Losa	249	DDAVP	10.9	NA
**2010**				
1. Alwani	79	Clinical SC LDDST UFC	20	16.5 (7–121)
2. Valassi	620	Morning SC UFC	13	66
**2011**				
1. Ammini	81	Clinical SC LDDST	18.5	34.8
2. Bou Khalil	127	Clinical SC UFC	21	NA
3. Storr	183	Clinical SC LDDST	21.4, micro 33.3, macro	NA
**2012**				
1. Ciric (133)	136	SC Clinical	9.7	108 (12–176)
2. Hassan-Smith	72	Clinical LDDST UFC	13.3	25.2 (15.6–37.2)
3. Honegger	83	Clinical SC LDDST UFC	7.4, micro 0, macro	37.0 (20–56)
4. Kim	54	SC LDDST UFC	47.4	57.2 (13–148)
**2013**				
1. Alexandraki	131	Clinical LDDST	24.4	65.1
2. Berker	90	Clinical SC LDDST	5.6	20.5 (20–35)
3. Lambert	346	Clinical SC UFC LDDST	10.8	69.6 (14.4–345)
4. Starke	66	Clinical SC UFC	NA	NA
5. Wagenmakers	86	Midnight SC LDDST UFC	16.1	42 (10–98)
**2014**				
1. Dimopoulou	120	Clinical LDDST UFC	34.1	54 (5–205)
**2015**				
1. Amlashi	224	Clinical UFC ODST LNSC	28	21.7 (3.1–54.0)
2. Aranda	35	Clinical Midnight SC LDDST UFC	65	28.8 (6–60)
3. Shin	50	Clinical Midnight SC LDDST UFC	18	NA
**2016**				
1. Chandler	276	Clinical ODST UFC	17	48
2. Sarkar	64	Clinical Midnight SC LDDST UFC	6.3	29
**2017**				
1. Espinosa-de-los-Monteros	89	UFC	22	48 (28.5–63)
2. Feng	341	UFC	2.42	NA (12–36)
3. Johnston	101	NA	7.2	NA
4. Shirvani	96	Clinical UFC ODST LNSC	21.9	24 (4–38)
**2018**				
1. Brichard	71	NA	18	36 (18–156)

*Adapted from Fleseriu et al. (12)*.

*SC, Serum Cortisol; UFC, 24-hour urinary free cortisol; ODST, Overnight dexamethasone suppression test; LNSC, Late-night salivary cortisol; NA, Not available; LDDST, Low-dose dexamethasone suppression test; ACTH, Adrenocorticotropic Hormone; DDAVP, 1-deamino-8-D-arginine vasopressin; Micro, Microadenomas; Macro, Macroadenomas*.

## Definition of Cushing's Disease Remission and Recurrence

While disease remission is defined by a low serum cortisol (SC) in the immediate postoperative period (2, 12, 14) and in some studies normal UFC and normal LNSC (13, 15), there is no clear established definition of CD recurrence. Notably, interpretation of studies evaluating postsurgical remission have multiple caveats, including, which criteria were used to define remission (adrenal insufficiency; AI, SC, UFC, overnight dexamethasone suppression test; ODST), timing of testing after surgery, perioperative administration of empirical glucocorticoids, presurgical use of cortisol-lowering medications, and time of follow up (16). In general, CD recurrence is manifested by a return of clinical symptoms and biochemical evidence of hypercortisolism. However, there is no consensus on which tests, or combination of tests and timing of tests for a definitive recurrence diagnosis. Various tests have been studied to assess an ability to predict, which patients in remission postoperatively will later experience recurrence, however, none have demonstrated sufficient accuracy (12, 17, 18).

## Clinical Picture: Symptoms and Clinical Findings in Recurrent Hypercortisolism

A 21-year-old male presents with hypertension, lower extremity weakness, poor wound healing, and central weight gain. There is face rounding, violaceous stretch marks, ecchymoses, and thinning of the skin. Laboratory evaluation reveals SC of 33.9 μg/dl, 24-h UFC of 272 μg/day (normal up to 45 μg/day), 1 mg DST is 19.1 μg/dl, and ACTH is 63 pg/ml. Pituitary MRI reveals a 5 mm pituitary adenoma. Inferior petrosal sinus sampling (IPSS) confirms a central source. The patient undergoes TSS and pathology confirms an ACTH positive pituitary adenoma. Post-operative serum cortisol is 3.3 μg/dl and ACTH is 18 pg/ml on day 1. Symptoms resolve, and the patient is considered in remission with AI requiring glucocorticoid replacement for 1 year; this is stopped when adrenal function is normal (confirmed by normal ACTH stimulation test). Three years post-operatively the patient experiences proximal weakness and central weight gain of 20 pounds with supraclavicular and dorsal fat deposition, poor concentration and fatigue.

### Question

Does this patient have recurrence or could they have pseudo-Cushing's (non-neoplastic hypercortisolemia) caused by stress, depression or sleep deprivation due to work-life balance?

## What Is the Next Best Step?

While CS has a variety of manifestations, recurrence of signs, and symptoms more specific for hypercortisolism such as easy bruising, wide violaceous stretch marks, osteopenia and thinning of the skin (19), as well as the subjective perception of the recurring symptoms by the patient should alert the clinician to begin a biochemical workup. Various tests to evaluate recurrence of hypercortisolism are described below.

## Biochemical Workup for Suspected Recurrence

### Post-surgical Serum Cortisol (SC) Levels in Cushing's Disease

Adrenal insufficiency postoperatively was previously interpreted as an indicator of long- term cure, but several studies have now shown that recurrence rates increase with longer follow-up even in patients with postoperative AI (20, 21). Studies that used postoperative SC cutoffs of <2 and 5 μg/dl found a recurrence rate of 3.1–4% with shorter follow up (mean 33–87.6 months), (14, 22) compared with 9–11% in studies with longer follow up (median 86.4; mean 92 months) (15, 20). Patients with SC > 2.0 μg/dl have 1.5 times greater odds of developing recurrence as compared to patients with levels <2.0 μg/dl (OR 2.5; 95% CI 1.12–5.52, *p* = 0.022) (23). While recent studies might support a nadir postoperative cortisol as a predictor of long-term “disease -free” with a positive predictor value (PPV) for remission of 90.5% when cortisol is <2 μg/dl and 80% when cortisol is <5 μg/dl (80%; 95%CI 66–94%) (1, 24), there is no cortisol value that can exclude all patients who will experience recurrence (12).

Furthermore, remission may be delayed in ~5.6% of patients after TSS, who remain hypercortisolemic and experience a cortisol decrease to normal after a median of 25 days (4–180 days) and some who become hypocortisolemic after a median of 8 days (4–150 days). However, a “delayed remission” group is significantly more likely to experience recurrence when compared with patients with immediate postsurgical remission (43 vs. 14%, *p* = 0.02) (25). Hameed et al. found that patients with postoperative SC >10 μg/dl are not likely to experience delayed remission (26). A postoperative cortisol <2 μg/dl and ACTH <5 pg/ml was found to have a PPV of 100% for remission, although no level predicted lack of recurrence, and ACTH/cortisol ratio did not predict the length of remission (26). Similarly, Costenaro's group found that SC nadir of ≤ 3.5 μg/dl within 48-h and ≤ 5.7 μg/dl within 10–12 days post-operatively predicted surgical remission with specificity and PPV of 100% (27).

Hypocortisolemia after surgery is still regarded as a marker of early remission, but cannot predict long-term remission; current clinical guidelines and disease state review recommend life-long clinical follow up in all patients (2, 12).

## Late Night Salivary Cortisol

Late night salivary cortisol has high sensitivity and specificity (90.0 and 91.8%, respectively) in the initial diagnosis of CS in the appropriate clinical setting (28), similar to midnight plasma cortisol (29).

**Figure 1:**
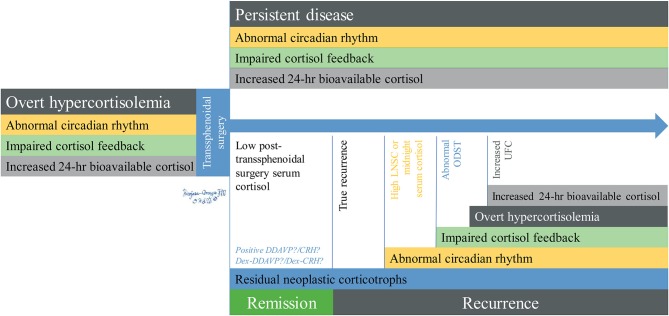
Chronological changes of recurrent hypercortisolemia in Cushing's disease. Overt hypercortisolemia is characterized by abnormal circadian rhythm which can be assessed by LNSC, impaired feedback shown by lack of suppression of ACTH/cortisol after dexamethasone, and increased levels of bioavailable cortisol measured by UFC. After TSS, patients with persistent disease will retain these abnormalities while they will resolve in the ones who experience remission, who will also have low post-TSS serum cortisol (remission) or normalized during the first ~25 days post-op (delayed remission). Residual neoplastic corticotrophs may be identified during remission or early recurrence by DDAVP/Dex-DDAVP and potentially by CRH/Dex-CRH. Abnormal circadian rhythm is the first abnormality that appears after recurrence, followed by impaired cortisol feedback, and finally by overt hypercortisolemia. DDAVP testing for hypercortisolemia is rarely performed in the United States. ACTH, adrenocorticotropic hormone; CRH, corticotropin releasing hormone stimulation test; DDAVP, desmopressin stimulation test; Dex-DDAVP, desmopressin stimulation test after low-dose dexamethasone suppression test; Dex-CRH, corticotropin releasing hormone stimulation test after low-dose dexamethasone suppression test; LNSC, late-night salivary cortisol; ODST, overnight dexamethasone suppression test; UFC, 24-h urinary free cortisol; TSS, transsphenoidal surgery.

Since circadian rhythm alterations are the first sign of hypothalamus-pituitary-adrenal axis dysfunction and precede defects in both negative feedback (evaluated by ODST) and hypercortisolemia (screened by 24-h UFC) (Figure 1) (30, 31), this test has been used for the initial diagnosis of both subclinical and overt presentation of CS with a sensitivity and specificity of 90% for the former (28) and 92–93% for the latter (32, 33), as well for identification of patients with non-neoplastic Cushing's (pseudo-Cushing's) syndrome (31).

Late night salivary cortisol should be the first choice in assessing CS recurrence (33). Nonetheless, a variety of pre-analytic factors may influence the results including method of obtaining saliva, sample contamination with blood, or vomit especially in the postoperative period, lack of saliva, timing compliance, and medication inference. Analytic assay issues have been reported with both direct immunoassay and liquid chromatography (less so with tandem mass spectrometry; LC/TMS). Direct immunoassay, including radioimmunoassay (RIA), manual enzyme-linked immunosorbent assay (ELISA) and automated electrochemiluminescent/electrofluorescent immunoassay (ELCIA/ELFIA), can cross-react with cortisone and synthetic steroids although they have the advantage of not being expensive, require less saliva volume, and are simple to perform. On the other hand, LC/TMS has minimal cross-reactivity and can identify sample contamination, but at a higher cost, requiring laboratory personnel expertise and a larger extraction of saliva volume (34, 35). Raff and Phillips (2019) reported a good correlation between LNSC measured by LC/TMS and the standard US Food and Drug Administration-cleared LNSC enzyme immunoassay in healthy adults, using smaller saliva volumes (50 μl) and measuring at normal bedtime, instead of late night sampling (36).

In a large study, postsurgical remission has been established with a LNSC cut-off of 1.9 nmol/l measured by enzyme immunoassay, with 94% sensitivity and 80% specificity. During follow up, a cutoff point of 7.5 nmol/l diagnosed recurrence with 75% sensitivity and 95% specificity a PPV of 92% and negative predictive value (NPV) of 80%. A lower cutoff point of 4.7 nmol/l increased the sensitivity (87%) and NPV (87%) at the expense of specificity and PPV (73 and 74%, respectively) (37). Other data on serial postsurgical LNSC using RIA also achieved specificity of 92.9% with 90% sensitivity (38).

Nonetheless, variability can be an important pitfall; a prospective study following 19 patients with confirmed CD found a great variability of LNSC measured by LC/TMS in all patients with early recurrence, presenting with maximal levels ranging from 1.55 to 15.5 × ULN, but all patients had at least two normal results and more than 50% of the tests performed in the same patient were normal (11). This suggests that LNSC also has some accuracy issues for diagnosing early recurrence of CD, although seems overall to be a better test than 24-h UFC (2, 12, 13, 17, 37).

## Dexamethasone Suppression Tests

Both the overnight 1-mg ODST and the 2-mg low-dose dexamethasone suppression test over 48 h (LDDST) evaluate the lack of feedback inhibition of corticotrophs by glucocorticoids and have good initial diagnostic accuracy, with LDDST slightly lower than that of ODST (2). While ODST is a common test performed for the screening of an initial CS diagnosis and LDDT an adjuvant screening test (2), there are no clinical studies specifically assessing these tests for their diagnostic yield for long-term remission or recurrence. ODST is expected to become abnormal in most cases after LNSC, but before 24-h UFC (17, 30). A study of 174 patients that underwent TSS found that a cortisol level <3 μg/dl on postoperative day 3 after ODST (early remission) predicted a 93% chance of remission at 5-year follow up (39). Current recommendations suggest a more stringent cutoff level (<1.8 μg/dL) (2, 12, 13) for both tests than the above mentioned study (previously a cutoff <5 μg/dl after ODST was considered normal).

## 24-Hour Urinary Free Cortisol

Urinary free cortisol is one test currently recommended for CS screening at initial diagnosis (2), but seems to lag in diagnosing early recurrence after TSS; a few studies report it as the last biochemical test with abnormal levels in this setting (mean time 50.6 months) (30, 37). Although UFC is a direct reflection of unconjugated and bioavailable cortisol in 24 h (19), interpretation of results may be difficult due to multiple patient factors such as increased water intake, collection volume and kidney function (40). Laboratory methodology may also be a factor (19, 40); RIA and electrochemiluminescence methods react to synthetic steroids and cortisol metabolites whereas high-pressure liquid chromatography and mass tandem spectrometry do not; however some drugs such as fenofibrate and carbamazepine may interfere with the assay (19, 41). Furthermore, intra-patient results can vary at ~50% in consecutive 24-h urine collections and this variability persists even after more than 2 collections are performed (41).

In a study of 50 patients with an initial CD remission after TSS and 15 with documented recurrence (by LNSC values), only 3 patients (20%) had an abnormal 24-h UFC (40). This was concordant with a study assessing early recurrence, finding a mildly elevated (1–2 × ULN) 24-h UFC in 39% of patients at recurrence diagnosis (38). A longer term study (median time of 53.5 months after TSS) found a low (68%) sensitivity of 24-h UFC although a high specificity (100%; NVP 78%) to establish recurrence at a cutoff point of 1.6 × ULN; adjusting to a stricter cutoff point (> 1.01 × ULN) provides the same sensitivity, but decreased specificity and NPV to 89 and 76%, respectively (37). The data confirms that using 24-h UFC alone might lead to missing the diagnosis of early recurrence of CD (2, 12, 13, 40).

More pitfalls of each of these particular tests have been previously reviewed in detail (2, 12, 13) and can be also found elsewhere in this special research issue.

## Adrenocorticotropic Hormone Levels

The role of ACTH in diagnosing either remission or recurrence is controversial. Adrenocorticotropic hormone has been studied as a predictor for remission and prognosis of recurrence following TSS for CD, either measured before treatment (42, 43), perioperatively (44), or during the immediate postoperative period (26, 45). These studies were performed with ACTH levels alone, in conjunction with dehydroepiandrosterone (DHEA) and DHEA sulfate (DHEAS), ACTH to cortisol ratio, or ACTH following suppression with betamethasone.

In one study (42), ACTH was significantly higher during the first 3 months postoperatively in patients with persistent disease compared to those with remission, however, there was no particular ACTH value to predict recurrence in the study (42). Furthermore, Hameed et al. showed in a large single center study that ACTH/cortisol ratio does not predict the length of remission (26). Although postoperative ACTH levels <5 pg/ml combined with serum cortisol levels <2 μg/dl predicted remission, specific cutoffs to predict recurrence were not established (26).

In another study, preoperative ACTH and ACTH/cortisol ratio were significantly higher in patients who experienced recurrence as compared to patients with sustained remission (43). Conversely, in another study all postoperative patients who later experienced recurrence had an ACTH value >20 ng/l and all those with sustained remission had <20 ng/l, with a statistically significant difference (44).

There have been concerns about assay interference and spurious results in ACTH (Immulite) assays, which can lead to misdiagnosis (46, 47). This has been described for immunoassays in the presence of heterophil antibodies and substances such as medications, metabolites or POMC, and/or ACTH fragments. Therefore, ACTH should not be used alone to diagnose remission or predict recurrence.

## Corticotropin-Releasing Hormone Stimulation Test After Dexamethasone Suppression

This test is currently the best option to distinguish non-neoplastic hypercortisolism (pseudo-Cushing's syndrome) from CS in patients who failed ODST during initial evaluation of hypercortisolism. First reported to have 100% sensitivity and specificity (48), subsequent studies reported a lower yield of diagnostic performance at the cutoff level of >1.4 μg/dl (49, 50) and test interpretation issues when performed with concomitant medication (51), with the possibility to increase specificity to 100% at a cutoff value of > 3.2 μg/dl, although sacrificing sensitivity (94%) (52). Another study found a threshold of 2.5 μg/dl al 15 min after CRH to have 90% sensitivity and specificity, although 15-min ACTH > 27 pg/ml had the greatest diagnostic accuracy (53). There is limited data of dexamethasone-CRH use in diagnosis of recurrence and optimal cutoff levels to rule out non-neoplastic hypercortisolism during recurrence are yet to be established.

## Corticotropin-Releasing Hormone Stimulation Test

Early CRH studies found that patients with a normal ACTH response to CRH compared to control subjects in the early postoperative period seem to have significantly greater recurrence rates than those with subnormal responses (54). In one study CRH-stimulated cortisol (5.4 ± 0.4 vs. 10.3 ± 1.7 μg/dl) and ACTH (23.5 ± 1.8 vs. 44.8 ± 8.5 pg/ml) were significantly higher in patients with recurrence than those with sustained remission (24). In another study a CRH stimulation test failed to predict remission in patients with exaggerated ACTH response when performed 7–10 days after surgery, however follow-up was insufficient to assess prediction of recurrence (55).

Again, potential assay interference should be taken into account when interpreting ACTH values obtained after CRH stimulation.

## Desmopressin Stimulation Test

Stimulation testing with 10 μg of 1-deamino-8-D-arginine vasopressin (desmopressin, DDAVP) was previously proposed as a method to diagnose CD (56) and distinguish the differential diagnoses of ACTH-dependent CS (sensitivity 83% and specificity 62%) (7, 57). Currently it has been proposed for ruling out non-neoplastic hypercortisolism, but may have lower sensitivity (75–90%) and specificity (90–92%) than CRH stimulation test after dexamethasone suppression (50, 58–60). Interestingly, a recent study found an ACTH peak of 71.8 pg/ml following DDAVP dose to have a higher diagnostic yield, with 90.8% sensitivity and 94.6% specificity, 95.3% PPV and 89.9% NPV. In this same study, an ACTH increment >37 pg/ml from baseline had 88% sensitivity, 96.4% specificity, 95.3% PPV, and 87% NPV (61).

Nine studies have reported the usefulness of a DDAVP test to detect early recurrence (56, 62–69). The use of the DDAVP test postoperatively can exploit its ability to detect the presence of residual neoplastic corticotroph cells, and hence a possible increased risk for relapse. The test was performed as soon as 4 days and as late as 6 months after TSS. Absolute cortisol increments of 7.0–7.4 μg/dl from baseline after DDAVP administration were found to have a NPV of 92%, specificity of 95%, PPV of 77%, and sensitivity of 68% for recurrence when data was combined by Vassiliadi et al. (7, 56, 62–66). Also, Vassiliadi et al. reported that a cortisol increment of ≥ 7.4 from baseline had a hazard ratio of recurrence of 24.7 (95% confidence interval, 10.6–448.5) at a median of 60 months, and that a loss of response to DDAVP posed a favorable prognosis for sustained remission (7, 66). A study assessing ACTH responsiveness set the cutoff level of 27 pg/ml increase from baseline as a positive test criterion for recurrence. Interestingly, in this study the reappearance of ACTH response to DDAVP preceded the recurrence of hypercortisolism by months to years (70).

## Desmopressin Stimulation After Dexamethasone Suppression

The performance of the DDAVP stimulation test has been also performed after dexamethasone suppression. An increase of > 50% on cortisol and ACTH levels had a sensitivity of 100% and specificity of 89% (71), and interestingly, the test was positive in some patients while they were still cortisol insufficient (71). The authors hypothesized that a positive test may identify patients at risk of recurrence and mandate closer monitoring, rather than need for treatment at that time.

Another study by the same group found that the combined test was more precise than DDAVP stimulation test alone to predict the lack of recurrence of CD with a NPV 100%, sensitivity 100%, specificity 71% and PPV 41% (63).

Theoretically it may be possible that the late appearance of ACTH or cortisol reactivity to DDAVP stimulation after initial remission may represent true recurrence, while early postoperative reactivity may indicate the persistence of tumoral corticotroph cells (7), however more research is needed.

Desmopressin is not used in the US neither for initial CD diagnosis nor for recurrence.

A summary of sensitivity and specificity of the above tests in diagnosis recurrence from various studies is shown in Table 2.

**Table 2:** Diagnostic accuracy of tests for Cushing's disease recurrence.

**Test**	**Cutoff level**	**Sensitivity (%)**	**Specificity (%)**	**PPV (%)**	**NPV (%)**	**Pros**	**Cons**	**References**
LNSC	7.5 nmol/l	75–90	92.9–95	92	80	In most patients abnormal earlier than ODST and/or UFC	Intra-patient variability May be normal despite recurrence	Amlashi et al. (37) Danet-Lamasou et al. (38)
24-h UFC	1.6 × ULN	68	100	NA	78	Direct reflection of bioavailable cortisol	~50% intra-patient variability Last to become abnormal	Amlashi et al. (37)
DDAVP	7.0–7.4 increments	68	95	75	92	First test to become positive in some studiesPredicts presence of tumoral corticotrophs	Dynamic testing Can become positive before clinical recurrence	Barbot et al. (62) Le Marc'Hadur et al. (63) Losa et al. (56) Romanholi et al. (64) Válero et al. (65) Vassiliadi et al. (66)
Dex-DDAVP	50% increase of ACTH	100	71–89	41	100	May be more accurate than DDAVP alone	Few studies assessing	Castinetti et al. (71) Le Marc'Hadur et al. (63)

*LNSC, Late-night salivary cortisol; UFC, 24-hour urinary free cortisol; DDAVP, Desmopressin stimulation test; Dex-DDAVP, Dexamethasone suppression and desmopressin stimulation test; PPV, Positive predictive value; NPV, Negative predictive value; NA, Not available; ODST: Dexamethasone suppression test; Sensitivity and specificity of this test for detecting recurrence are not well studied; ACTH, Adrenocorticotropic hormone*.

## Assessing Remission in Patients on Preoperative Medical Therapy

Although surgery is the most common first-line treatment for all CS types (78%), it is estimated that around 20% of patients need to take preoperative medical therapy and ~2% are treated with medical treatment only (72). Interestingly, clinical improvement after surgery is less likely to be reported by patients with presurgical medical therapy than those without previous treatment (76 vs. 83%, *p* = 0.04), but the former group was more frequently reported to have postsurgical cortisol levels within the normal range than the later (23 vs. 13%, *p* = 0.006). Furthermore, postsurgical low or undetectable cortisol levels were more frequently found in patients without previous medical therapy (69 vs. 60%, *p* = 0.01), with higher odds early after surgery [OR 0.48 (95% CI 0.30–0.76); *P* = 0.002]. There was no difference in the prevalence of postsurgical morbidities or remission rates between presurgical treated and untreated patients (72). One can envision that assessing recurrence in these patients who were on medical therapy before surgery would be more complicated, especially in cases with no clear-cut features of remission either, however, more data is needed.

## Role of Localization Tests After Confirmation of Hypercortisolemia Recurrence

In most patients, recurrence of hypercortisolemia has the same etiology as initial diagnosis. However, in absence of a clear-cut diagnosis (pituitary pathology with ACTH staining, postoperative AI), reestablishing the source of ACTH excess is important before any further treatment.

### Inferior Petrosal Sinus Sampling

Inferior petrosal sinus sampling is the most accurate method to locate the source of ACTH-dependent CS (19). If not performed during initial workup before the first surgery and if no pathological confirmation of an ACTH-secreting tumor is available, IPSS should be performed to confirm the ACTH-secreting source and guide further workup (2). Repeat IPSS may be considered if there is reasonable suspicion of an inaccurate or equivocal former procedure, which can occur up to 10% of cases (73, 74) as well in the case where a second ACTH-secreting tumor is suspected, which is extremely rare.

### Pituitary Imaging to Detect Recurrent Tumors

Since most CD cases are secondary to an ACTH-secreting microadenoma, contrast-enhanced pituitary magnetic resonance imaging (MRI) remains the study of choice for surveillance to detect recurrent or growing residual tumors. New evidence of tumor or growth of previous residuals on MRI should prompt biochemical evaluation in patients in clinical remission (12).

**Figure 2:**
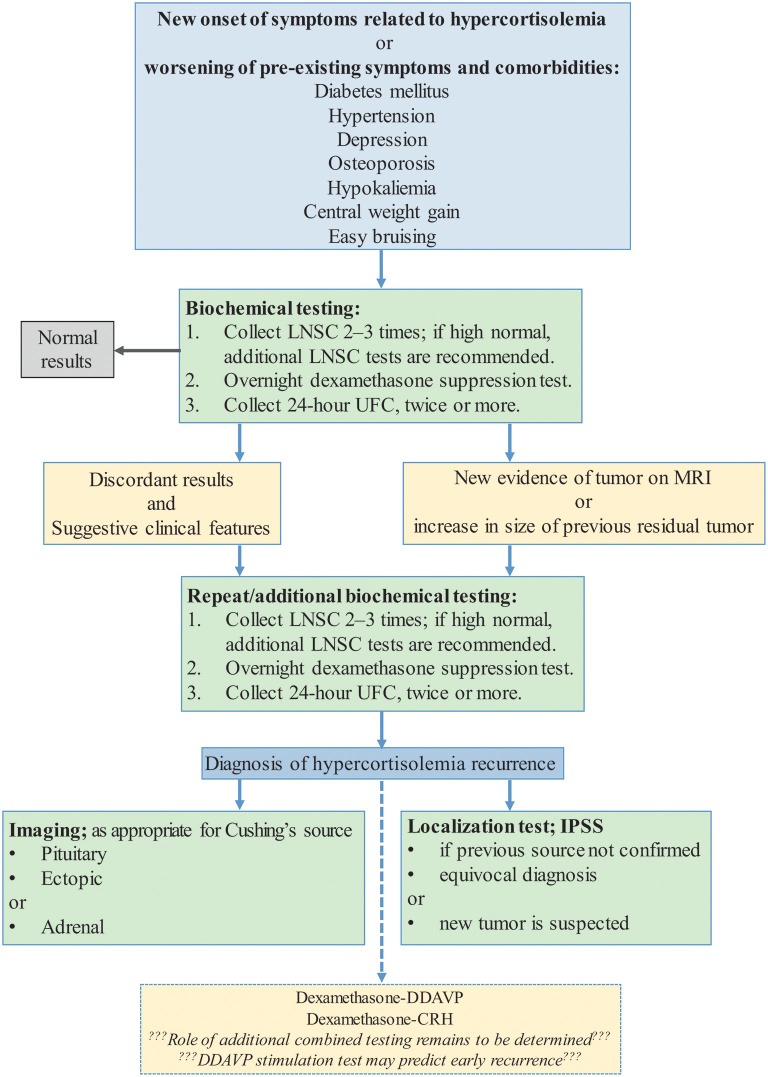
Clinical suspicion of recurrent Cushing's Syndrome (2, 12, 78). DDAVP, desmopressin stimulation test; Dex-DDAVP, desmopressin stimulation test after low-dose dexamethasone suppression test; Dex-CRH, corticotropin releasing hormone stimulation test after low-dose dexamethasone suppression test; LNSC, Late-night salivary cortisol; ODST, overnight dexamethasone suppression test; UFC, 24-h urinary free cortisol.

Positron emission tomography (PET)-CT and PET-MRI have been also recently proposed as potential methods to locate the secreting tumor, but diagnostic yield and cost-effectiveness is yet to be evaluated (75, 76). Patients with CD and adrenal tumors may develop ACTH-independent hypercortisolism and “recur” due to adrenal CS (77). Suppressed ACTH levels in these cases will aid in the differential diagnosis. A flowchart depicting suggested testing is shown in Figure 2.

## Conclusions

Diagnosis of CD recurrence is challenging especially early on and must be distinguished from non-neoplastic hypercortisolemia. Late night salivary cortisol is the first test to reveal abnormal results, ODST the second, while 24-UFC does not increase above normal until later in recurrence course. Existing tests used for CD initial diagnosis need new cut-off values for establishing recurrence and/or to predict a possible recurrence in patients with remission after surgery. Clinical features and evidence of tumor regrowth on MRI are additional factors that should prompt suspicion for recurrence of CD and warrant biochemical evaluation. Further studies are needed to determine frequency of testing in patients with remission and an ideal sequence of testing in patients to establish recurrence. A search for and research on new accurate diagnostic tests and/or tools to accurately determine recurrence is warranted. After a clinical and biochemical diagnosis of recurrence, therapy should be individualized and physicians and patients need to balance benefits with possible adverse effects of each type of treatment.

## Author Contributions

JH-A, EV, SM, and MF have made substantial contributions to the conception or design of the work, the acquisition, analysis or interpretation of data for the work, drafted the work, critically revised it for important intellectual content, provided approval for publication of the content, and agreed to be accountable for all aspects of the work in ensuring that questions related to the accuracy or integrity of any part of the work are appropriately investigated and resolved.

### Conflict of Interest

MF disclosures: Principal investigator with research funding to OHSU from Novartis, Millendo, Strongbridge, and has received occasional scientific consulting fees from Novartis and Strongbridge. The funders were not involved in the study design, collection, analysis, interpretation of data, the writing of this article or the decision to submit it for publication. The remaining authors declare that the research was conducted in the absence of any commercial or financial relationships that could be construed as a potential conflict of interest.
